# UBC9 mediates mitophagy to attenuate oxidative stress by regulating SUMOylation of PINK1 in the Parkinson’s disease progression

**DOI:** 10.1007/s10565-025-10126-3

**Published:** 2025-12-06

**Authors:** Jian Liu, Ge Jia, Yu Zhou, Junmei Zhang, Yanjin Wang, Yuxiang Cai

**Affiliations:** 1 Department of Neurosurgery, Xiangya Hospital, Central South University, No. 87, Xiangya Road, Changsha, 410008 Hunan Province P. R. China; 2 Department of Neurosurgery, Xiangya Hospital, Central South University, Jiangxi (National Regional Center for Neurological Diseases), Nanchang, 410008 Jiangxi Province P. R. China

**Keywords:** UBC9, PINK1, Mitophagy, Oxidative stress, Parkinson’s disease

## Abstract

**Background:**

Parkinson’s disease (PD) is a neurodegenerative disease characterized by progressive loss of dopaminergic neurons. UBC9 is related to the formation of several cancers. Nevertheless, the function of UBC9 in PD and the potential mechanisms are vague.

**Methods:**

MPP⁺-induced SH-SY5Y cells and MPTP-treated C57BL/6 mice were applied to induce PD models. Cell viability, proliferation and apoptosis were measured using CCK-8, EdU and Annexin V/PI staining, respectively. JC-1 staining and fluorescent probes DCFH-DA were employed to measure mitochondrial membrane potential and ROS production. The SOD, GSH and MDA content were determined by the commercially kits. SUMOylation of PINK1 were predicted by SUMOplot and verified by co-IP/Western blot. Mitophagy-related proteins, SUMO enzymes, and TH were analyzed by qRT-PCR/Western blot. LC3 expression was detected via immunofluorescence staining. Transmission electron microscopy was performed to detect autophagy. MPTP-induced brain injury was evaluated using Nissl staining, IHC and TUNEL assay. Motor function was observed via open field test and pole test.

**Results:**

PINK1 and UBC9 were low-expressed in MPP^+^-induced SH-SY5Y cells. UBC9 mediated PINK1 SUMOylation. UBC9 overexpression promoted cell viability and reduced cells apoptosis in MPP^+^-stimulated SH-SY5Y cells, which was reversed after PINK1 silence or CsA treatment. Moreover, UBC9 overexpression counteracted MPP^+^-induced mitophagy, and oxidative stress. However, these findings were reversed by CsA or PINK1 silencing. PINK1 bound SUMO1 at the K522, K363 and K193 sites, further regulating cells viability and apoptosis. In MPTP-treated mice, UBC9 overexpression alleviated mitochondrial dysfunction and motor deficits via PINK1 SUMOylation.

**Conclusion:**

UBC9 mediated mitophagy to attenuate MPP^+^/MPTP-induced neurotoxicity and oxidative stress by regulating PINK1 SUMOylation, suggesting that UBC9 may play a preventive role in PD progression.

## Highlights

- UBC9 enhanced PINK1 stability by promoting SUMOylation of PINK1 at the K522R/K363R position.
- UBC9 regulated MPP^+^-induced SH-SY5Y cells proliferation and apoptosis via PINK1.
- UBC9 alleviated MPTP-induced motor dysfunction and oxidative stress via PINK1-mediated mitophagy.

**Graphical abstract:**

## Graphical abstract

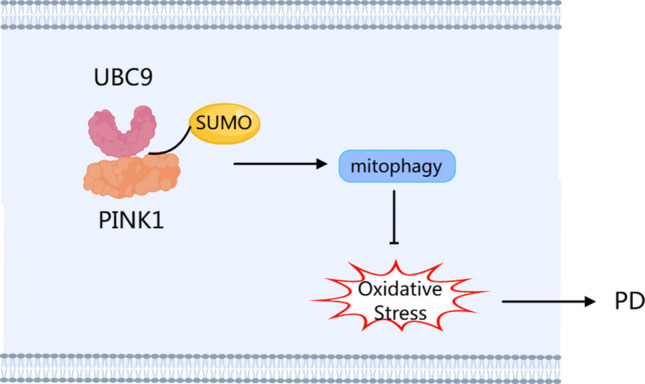

## Introduction

Parkinson’s disease (PD) is a frequent neurodegenerative disease with high morbidity, disability and mortality. It is featured with degenerative death of dopaminergic neurons and accumulation of Lewy bodies (Okitsu et al. 2023). The global burden of disease research reported in 2021 revealed that 8.5 million people suffer from PD worldwide, which severely reduces the quality of PD patients’ life (Xu et al. 2024). Although the exact pathogenesis of PD are unknown, several elements including oxidative stress, mitochondrial dysfunction, activation of the apoptotic cascade and neuroinflammation were shown to be key factors in the pathogenesis of PD (Simon et al. 2020). At present, dopamine replacement (levodopa) is the widely recognized treatment to alleviate the symptoms of PD, while it cannot block the progression of the disease (Wang and Shih 2023). Thus, an in-depth research on the pathogenesis of PD is essential to find effective preventive drugs.

Intramitochondrial Ca^2+^ overload may enhance intracellular reactive oxygen species (ROS) production, thereby damaging the related cells. A large body of evidence demonstrated that PD-associated cellular damage is closely related to mitochondrial dysfunction (Picca et al. 2021). Impaired mitophagy leads to the accumulation of dysfunctional mitochondrial and α-synaptic nuclear proteins, which in turn results in abnormal neuronal function and death (Li et al. 2022). Study reported by Prasertsuksri P et al. indicated that andrographolide exerts an obvious effect on neurotoxin MPP^+^ induced SH-SY5Y cells by activating mitochondrial autophagy and antioxidant activity (Prasertsuksri et al. 2023). Additionally, Chen et al. provided evidence that dexmedetomidine may enhance PINK1/Parkin-induced mitophagy to ameliorate mitochondrial roles via activating AMPK in PD (Chen et al. 2022). To date, the molecular mechanisms by which mitochondrial dysfunction regulates PD remain unclear.

PINK1 and Parkin are markers of mitochondrial dysfunction leading to mitophagy in neurodegenerative diseases, including PD (Quinn et al. 2020). PINK1 is a serine/threonine kinase located in the outer membrane of the mitochondria, which recruits Parkin, an E3 ubiquitin ligase, and binds to the autophagy-related protein LC3 to initiate mitochondrial dysfunction. Inactivated PINK1 disrupts the mitophagy process, causing the accumulation of impaired mitochondria, enhanced oxidative stress, and neuronal degeneration in PD patients (Malpartida et al. 2021). Although the important role of PINK1 in the PD progression has been confirmed, the exact mechanisms of its regulation and protein stability have not been fully elucidated. Therefore, exploring the regulation of PINK1 stability is important for understanding the pathological mechanism of PD.

Small ubiquitin-like modifier (SUMO) is a protein modification process, which mediates protein stability, localisation and transcriptional activity. During SUMO modification, SUMO proteins are attached to lysine residues of target proteins via E1 activating enzymes (SAE1/UBA2), E2 binding enzymes (UBC9 or UBE2I) and E3 ligases, thus regulating protein stability and protein–protein interactions (Qin et al. 2021). Researches have found that SUMO modifications play key roles in a variety of biological and pathological mechanisms (Sahin et al. 2022). UBC9 is the most critical SUMO E2-binding enzyme involved in various cellular processes including nuclear translocation, transcriptional regulation and apoptosis. Verma et al. found that UBC9 protected dopaminergic cells from cytotoxicity and increased the α-synuclein stability in PD (Verma et al. 2020). However, it is still unknown whether UBC9 was involved in mitochondrial degradation in PD.

Therefore, we hypothesized that UBC9 may regulate mitophagy to alleviate oxidative stress through regulating SUMOylation of PINK1 in PD progression. Our investigation elucidated the neuroprotective role of UBC9 in the MPP^+^-stimulated SH-SY5Y cells and MPTP-treated PD mice, as well as explored the interaction of UBC9 with oxidative stress and mitophagy.

## Materials and methods

### Cell cultivate and treatment

The SH-SY5Y cells were procured from the ATCC (Manassas, VA, USA) and incubated in RPMI-1640 medium (Gibco, USA) containing FBS (Gibco, USA) and penicillin/streptomycin (Gibco, USA) with 5% CO_2_ at 37 °C. Cells were subjected to 2 mM MPP^+^ (HY-W008719, MedChemExpress) for 24 h to construct an in vitro PD cells model. Following MPP^+^ stimulation, cells were treated with mitophagy inhibitors (5 nM CsA, HY-B0579, MedChemExpress) for 24 h.

### Cell counting Kit-8 (CCK-8) assay

Cell Counting Kit-8 (CK04, solarbio, Beijing, China) was employed to determine the cells viability. SH-SY5Y cells were plated in 96-well plates and incubated at 37 °C for 24 h. Next, 10 μL of the CCK-8 solution was added to each well, followed by an additional incubation of 2 h at 37 °C. Finally, the optical density at 450 nm was detected using a microplate reader (Molecular Devices, USA).

### EdU assay

The proliferation of SH-SY5Y cells was assessed following the direction of SF594 EdU assay kit (CA1174-B, Solarbio). SH-SY5Y cells (4 × 10^3^ cells/well) were planted in 96-well plates for 24 h. Subsequently, the cells were incubated with EdU solution, washed with PBS, and fixed using 4% paraformaldehyde. Next, 0.5% Triton X-100 was introduced into each well and incubated for 10 min and apollo staining solution was treated for 30 min. Hoechst 33,342 solution was applied for DNA staining. Images were analyzed and visualized under EVOS M5000 Fluorescence Imaging System (Thermo Fisher Scientific).

### Cell transfection

PINK1-mut (K522R, K363R, K193R) were generated via the QuikChange Lightning Site-Directed Mutagenesis Kit (210518, Agilent). The overexpression plasmids including pcDNA3.1-UBC9 (oe-UBC9) and shRNA sequence targeting PINK1 (sh-PINK1), as well as their negative controls were acquired from Gene Pharma (Shanghai, China). SH-SY5Y cells (5 × 10^4^ cells/well) were cultured into 6-well plates and cell transfection was conducted by Lipofectamine® 3000 reagent (Invitrogen, USA) for 24 h following the manual. For overexpressing UBC9, UBC9 cDNA were used to amplify the coding region of UBC9 by PCR. The UBC9 PCR product was inserted into the pcDNA3.1 vector with a BamHI and EcoRI restriction (Thermo Fisher Scientific). For silencing PINK1, PINK1 cDNA was amplified with PCR and cloned into a PGMLV-hU6-MCS-CMV-ZsGreen1-PGK-Puro-WPRE vector. The shRNA sequence was PINK1: 5’-GCAUUCAGUUCCAGAACUATT-3’.

### Flow cytometry

The apoptosis of SH-SY5Y cells was assessed by Annexin V-FITC Apoptosis Detection Kit (C1062L, Beyotime). Cells were cultivated until reached 80% confluence. Next, the cells were digested with trypsin, dyed in dark with 5 μL FITC Annexin-V and 5 μL PI in line with the manufacturer's protocol. At last, the apoptotic cells were measured on a BD Biosciences FACS Calibur system (San Jose, CA).

### Transmission electron microscopy

For TEM analysis, cells (2 × 10^6^) were harvested and washed three times with PBS. Then the samples were fixed at room temperature in 1% osmium tetroxide (OsO_4_) for 2 h, followed by dehydration through a series of progressively diluted ethanol solutions. Subsequently, the samples were infiltrated and embedded in LR White resin (Santa Cruz Biotechnology). Ultrathin Sects. (60 nm) were prepared using an ultramicrotome, stained with uranyl acetate and lead citrate, and examined under a transmission electron microscope (Tecnai G2, FEI).

### Reactive oxygen species determination

The ROS quantification was carried out via ROS Assay Kit (S0033S, Beyotime). Briefly, SH-SY5Y cells were cultivated in 96-well plates and subsequently stimulated by 1 μM DCFH-DA at 37˚C for 12 h. Following incubation, cells were washed with PBS to eliminate any residual substances. Subsequently, the fluorescence intensity was measured at Ex488/Em525nm using a fluorescence spectrometer (Perkin Elmer, Waltham, MA).

### JC-1 staining

The mitochondrial membrane potential was determined according to the manufacturer's instructions of MitoProbe™ JC-1 Dye (T3168, Thermofisher). When SH-SY5Y cells were cultivated until reached 80% confluence, each sample was treated with JC-1 dye for 30 min at 37 °C.. After the JC-1 staining working solution was moved, fluorescence of mitochondrial membrane potential was observed using a ZEISS microscope (Germany).

### Measurement of SOD, CAT, GPX and MDA activity

SH-SY5Y cells (5 × 10^4^ cells/well) were planted into 96-well plates. Subsequently, the SOD, CAT, GPX and MDA activity in substantia nigra of mice and SH-SY5Y cells were checked by Superoxide Dismutase Assay Kit (S0060, Beyotime), Catalase (CAT) assay kit (A007-1–1, njjcbio), Cellular Glutathione Peroxidase Assay Kit (S0056, beyotime) and Lipid Peroxidation (MDA) Assay (ab118970, Abcam) following the manufacturer’s direction.

### Co-IP assay and SUMOylation analysis

Co-immunoprecipitations were conducted to evaluate SUMOylation of PINK1 according to the Protein A/G Magnetic Co-IP/IP Kit (K1309, APExBIO, Texas, USA). Cells transfected with plasmids containing SUMO1 and Flag tagged PINK1, PINK1-K522R, PINK1-K363R, PINK1-K193R, oe-UBC9 or sh-UBC9 were lysed and centrifuged to obtain cell lysates. Then cell lysates were cultivated with protein A beads for 3 h at 4 °C. Next, they were centrifuged to remove the Sepharose beads, and 5 μg of indicated antibodies were cultivated in the prewashed lysate overnight at 4 °C. After that, sepharose protein A beads were incubated with sample for another 4 h to catch the antibody-antigen complex. The mixture was centrifuged and washed with TBST to throw away the supernatant, and the bound proteins were determined using Western blot with the appropriate antibodies.

### Cycloheximide chase assay

To assess the stability of PINK1 protein, a cycloheximide (CHX) chase assay was performed. Cells were treated with CHX at a final concentration of 100 μmol/L. After the cell lysates were collected at specified intervals (0, 1.5, 3.0, 4.5, 6.0, and 7.5 h), the protein levels of PINK1 were analyzed by Western blotting, with β-actin serving as an internal control.

#### qPCR analysis

Total RNA was separated from SH-SY5Y cells by TRIzol® reagent (EP013, ELK Biotechnology). HiScript III qRT SuperMix (R323, Vazyme) was conducted for reverse transcription. Then, real-time PCR was performed by ChamQ Universal SYBR qPCR Master Mix (Q711, Vazyme) with CG Real Time PCR (Heal Force). The relative mRNA levels (SAE1, SAE2, UBC9, SENP1, SENP3, SENP5 and PINK1) were detected by the 2 − ∆∆Ct method and GAPDH was considered as control gene. Primers are presented in Table 1.

**Table 1:** Primers sequence

Gene	Forward Primer	Reverse primer
SAE1	5'-TGGAGCAGTGAGAAAGCAAAG-3'	5'-GGAAGCAGGTCAGGACTAATAC-3'
SAE2	5'-CACAGGTTGCCAAGGAA-3'	5'-GACACTCATAACACTCGGTCA-3'
UBC9	5'-GTCCTCCACCTGTCCGCTAC-3'	5'-TCTTGCCAAACCAATCCCT-3'
SENP1	5'-ATCAGGCAGTGAAACGTTGGAC-3'	5'-GCAGGCTTCATTGTTTATCCCA-3'
SENP3	5'-GGCAGAATAATGACAGTGAC-3'	5'-AGTGACACAGCTCCTTGT-3'
SENP5	5'-TGCTAGATCACCTCGTCTTCA-3'	5'-AGTGCTTAGTGGTTTTCATGATA-3'
PINK1	5'-GACCTCAAGTCCGACAACA-3'	5'-TGCCACCACGCTCTACAC-3'
GAPDH	5'- GGACCTCATGGCCTACATGG −3'	5'- TAGGGCCTCTCTTGCTCAGT −3'

### Western blot assay

Total protein extraction of brain tissues and SH-SY5Y cells was conducted by RIPA Lysis Buffer (ThermoFisher Scientific, USA) and analyzed by BCA Protein Quantification Kit (AS1086, ASPEN). Equal samples were subject to 12% SDS-PAGE and transferred onto PVDF membranes (IPVH00010, Millipore), followed by blocking with specific primary antibodies, including anti-PINK1 (DF7742, Affbiotech, 1:1000), UBC9 (ab75854, Abcam, 1:1000), Parkin (AF0235, Affbiotech, 1:1000), LC3 (AF5402, Affbiotech, 1:1000), p62 (ab109012, Abcam, 1:10,000), SUMO1 (ab32058, Abcam, 1:1000), COX IV (ab202554, Abcam, 1:2000), Flag (ab205606, Abcam, 1:1000), TH (AF6113, Affbiotech, 1:1000) and GAPDH (60,004–1-Ig, Proteintech, 1:50,000) overnight at 4 °C. Next, the membranes were incubated with secondary antibody (S0001, Affinity Biosciences, 1:5000). At last, the protein bands were detected with ECL reagent (AS1059, ASPEN).

### Animals and treatment

24 male C57BL/6 mice (10–12 weeks old) were provided by the Beijing Vital River Laboratory Animal Technology Co., Ltd.. Subsequently, these mice were housed and acclimated in a SPF grade animal experimental facility at 25° C with a standard light dark cycle of 12 h. Moreover, the ambient temperature stringently controlled at 22 ± 2 °C and the relative humidity stabilized within 40%−60%. The animals were divided into four groups (n = 6). Sham group, MPTP group, MPTP + AVV-oe-NC group and MPTP + AVV-oe-UBC9 group. The full-length mouse UBC9 cDNA (Gene ID: 22196) was cloned into the AAV plasmid under the control of the human synapsin (hSyn) promoterto generate the pcAAV-UBC9 construct. As a control, the empty vector was used to produce the AAV-control adenovirus. Mice in sham group received normal saline solution. MPTP (25 mg/kg/day, HY-15608, MedChemExpress) was injected intraperitoneally for 5 days along with AVV-oe-NC or AVV-oe-UBC9. All animal experiments were approved by the Ethics Review Committee of Xiangya Hospital, Central South University. (2024101736).

### Open field test

The open field test can accurately evaluate the overall performance status of motor deficits in PD mice. In the specific experimental setup, the open field area consists of a square box (50 × 50 cm) and a fence (40 cm tall). Mice were placed alone in the exact center of the box and given several minutes to gradually adapt to this brand-new environment. Subsequently, video recording equipment was used to record the behavioral activities of the mice for 5 min.

### Rotarod test

The motor coordination of mice was assessed by rotarod test. Before the formal experiment begins, the mice were placed on the stationary rotarod apparatus to allow them to adapt to the environment. Then, the rotation speed of the rotarod apparatus is set to 20 rpm within 1 min. The mice are placed on the rotating rotarod one by one, and timing starts simultaneously. The situation of the mice walking on the rotarod is observed, and the time from when each animal starts walking on the rotarod until it falls off is recorded.

### Imunohistochemistry

The brain tissues were obtained and fixed with 4% paraformaldehyde. After dehydration, 2–3 μm paraffin sections were cut from the fixed brain tissues. After deparaffinization and hydration, sections were treated with 3% H_2_O_2_ for 15 min and incubated with primary antibody against TH (AF6113, Affbiotech, 1:100) at 4 °C overnight. Then the sections were treated with corresponding secondary antibody, stained with diaminobenzidine (DAB) and counterstained with hematoxylin (H9627-25G, Sigma). Images were obtained by microscopy (Olympus, Japan).

### Nissl staining

For Nissl staining, the brain tissues were harvested, fixed in 4% paraformaldehyde, they were dehydrated and paraffin-embedded. Subsequently, the embedded brain tissue was coronally sectioned at a thickness of 20 μm. The sections were deparaffinized in xylene, hydrated in gradient ethanol, and then stained with Nissl staining solution (C0117, Beyotime) for 15 min. After that, anhydrous ethanol was employed for rapid dehydration. The sections were washed and sealed. The Nissl-stained sections were photographed under a microscope to observe the Nissl bodies of nigral dopaminergic neurons. Image J was utilized to analyze the average gray values of the Nissl-stained images.

### Immunofluorescence

Specimens fixed with formaldehyde were embedded in paraffin and sliced into sections with a thickness of 4 μm. The sections were deparaffinized using xylene and then rehydrated through a series of alcohol solutions with gradually decreasing concentrations. Next, the sections were incubated with LC3 (AF5402, Affbiotech, 1:200) at 4 °C overnight, then incubated with a secondary antibody (ab150077, Abcam, 1:200) for 30 min. The cell nuclei were stained with DAPI (C0065, Solarbio). Cells were analyzed under the inverted microscope (Olympus, Japan).

### Statistical Analysis

The values were given as mean ± standard deviation (SD). Differences among groups were analyzed using one-way analysis of variance (ANOVA) and student’s t-test. All experiments were conducted in triplicate. All statistical analyses were conducted by GraphPad Prism 8.0 software (La Jolla, CA, USA). A P < 0.05 level was considered statistically obvious difference.

## Results

### UBC9 regulated MPP^+^-stimulated SH-SY5Y cells viability and apoptosis

**Fig. 1:**
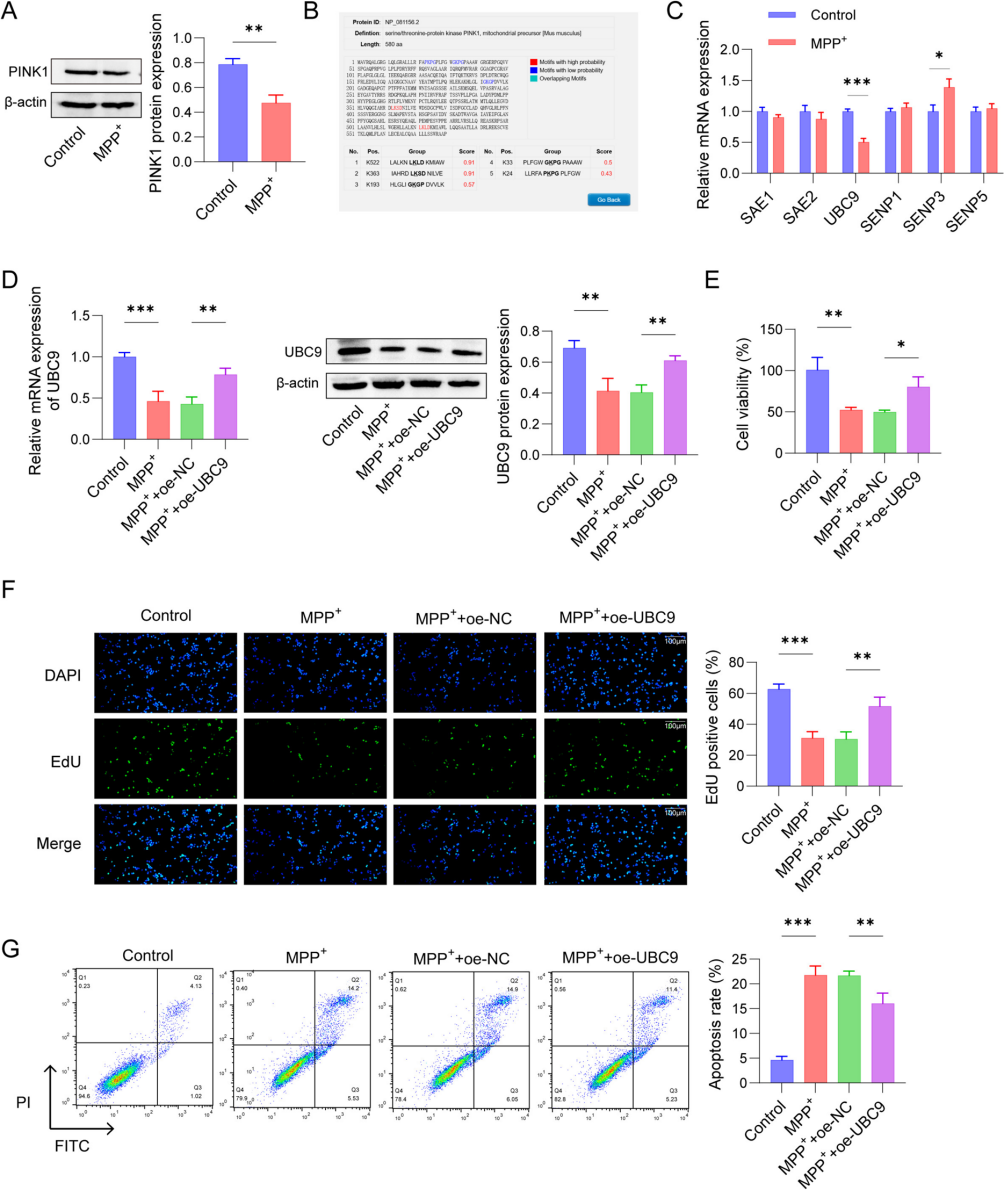
Effects of UBC9 on MPP^+^-treated SH-SY5Y cells viability and apoptosis. (**A**) PINK1 expression in MPP^+^-stimulated SH-SY5Y cells was measured using Western blot. (**B**) The SUMO modification site(s) in PINK1 were predicted by SUMOplot Analysis Program (https://www.abcepta.com/sumoplot). (**C**) qRT-PCR was applied to measure the mRNA levels of SAE1, SAE3, SENP1, SENP3 and SENP5. (**D**) UBC9 expression in SH-SY5Y cells treated with MPP.^+^ with/without transfected with oe-NC or oe-UBC9 was determined by using qRT-PCR and western blot. (**E**) Cell viability was measured using CCK-8 assay. (**F**) EdU assay was applied to detect cell proliferation ability. (**G**) Flow cytometry for SH-SY5Y cells apoptosis. *n* = 3, *p < 0.05, **p < 0.01, ***p < 0.001

Firstly, SH-SY5Y cells were induced by 2 mM MPP^+^ for 24 h to reveal the roles of PINK1 in neuronal cells. We observed that PINK1 expression was signally declined in MPP^+^-stimulated SH-SY5Y cells (Fig. 1A). Next, we predicted the SUMO modification site(s) in PINK1, two lysine residues were identified using the SUMOplot Analysis Program (Fig. 1B). Moreover, the levels of SUMO-related enzymes were explored. As presented in Fig. 1C, UBC9 was dramatically reduced and SENP3 was up-regulated in the SH-SY5Y cells after MPP^+^ induction, compared to the control group. Nevertheless, there were no obvious changes in SAE1, SAE3, SENP1, and SENP5 expressions, as evaluated by qRT-PCR. After stimulating cells with MPP^+^ or MPP^+^ + oe-NC, UBC9 mRNA and protein expression were decreased, as opposed to the control group, whereas an increase was displayed after up-regulating UBC9 (Fig. 1D). Moreover, the CCK-8 result revealed that cell viability in the MPP^+^ group was memorably inhibited, while overexpression of UBC9 presented an opposite result (Fig. 1E). The EdU assay and flow cytometry also verified that MPP^+^ induction decreased EdU-positive cells and elevated apoptosis rates, as opposed to control group, which was reversed by UBC9 overexpression (Fig. 1F-1G). These results reflected that UBC9 regulated MPP^+^-stimulated SH-SY5Y cells proliferation and apoptosis.

### UBC9 regulated the proliferation and apoptosis of MPP^+^-induced SH-SY5Y cells to alleviate oxidative stress by modulating mitophagy

**Fig. 2:**
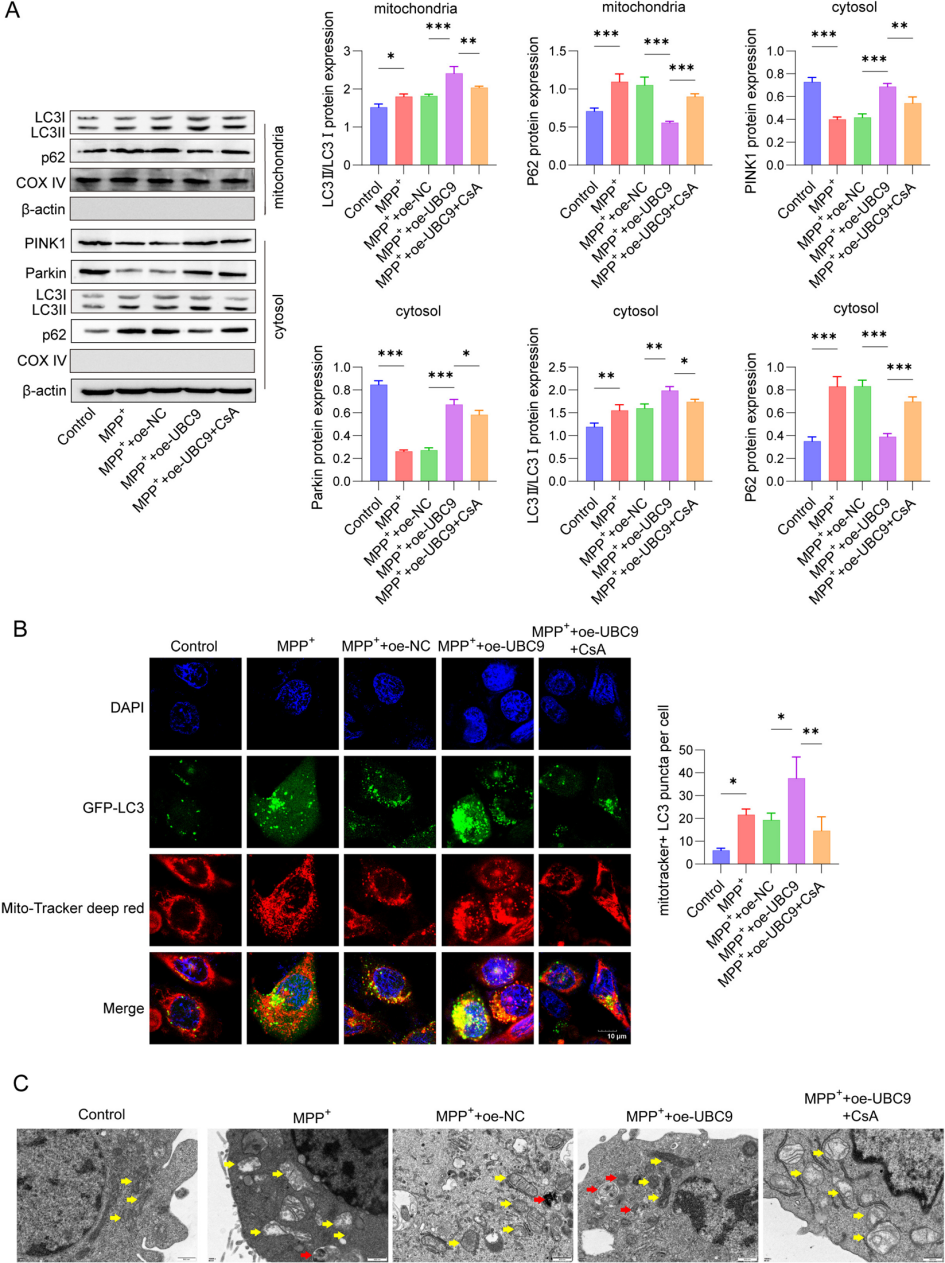

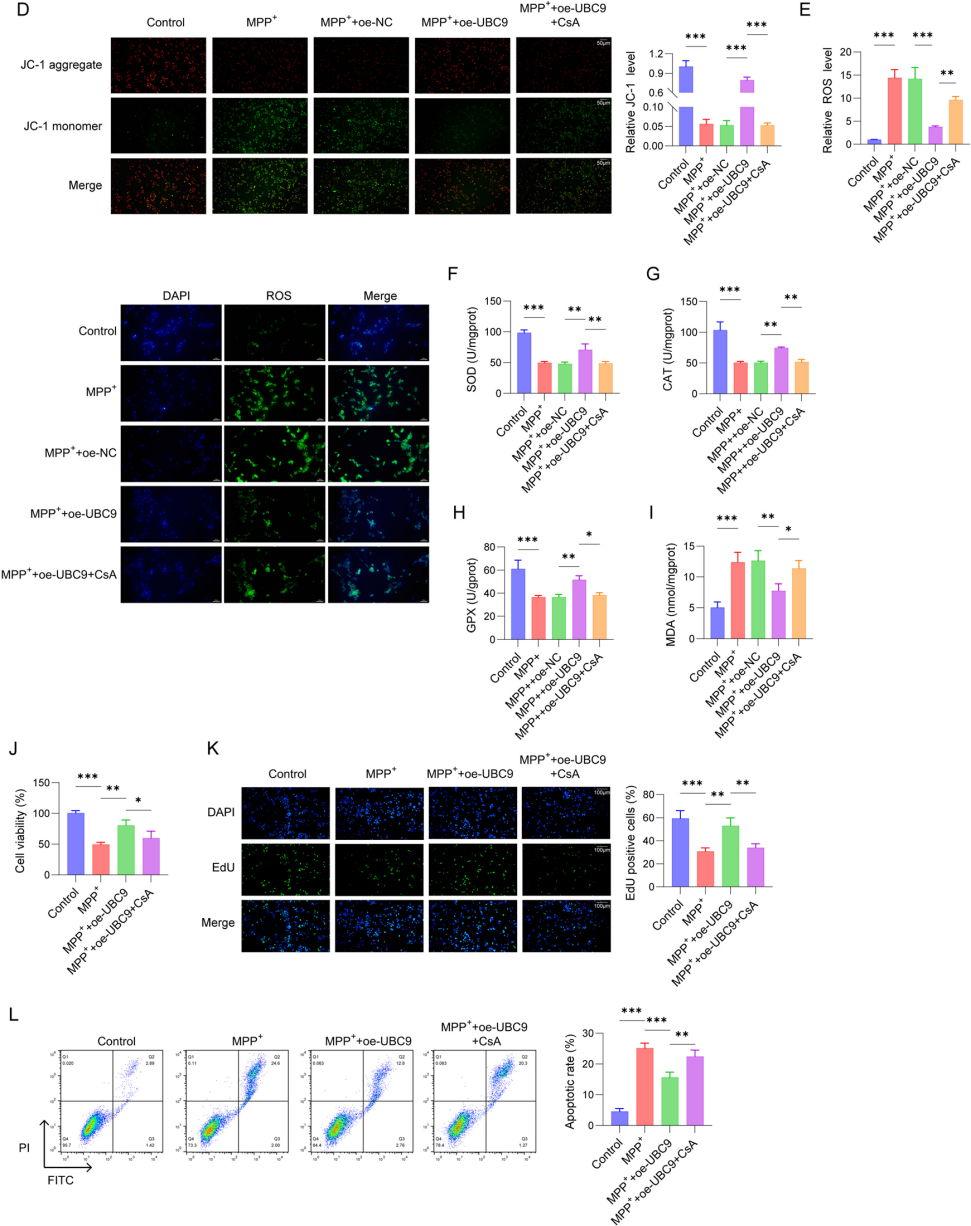
Effect of UBC9 on MPP^+^-induced SH-SY5Y cells viability, apoptosis, oxidative stress and mitophagy. SH-SY5Y cells transfected with oe-NC or oe-UBC9 were treated with 2 mM MPP.^+^ for 24 h, or stimulated with 5 nM CsA for 24 h. (**A**) Western blot for autophagy-related proteins. (**B**) Representative immunofluorescence images of LC3. (**C**) Representative TEM images of mitochondria. (**D**) The mitochondrial membrane potential changes were assessed using JC-1 staining. (**E**) DCFH-DA staining was performed to detect ROS generation. (**F**-**I**) Enzyme activities of SOD, CAT, Gpx and MDA were determined. (**J**) Cell viability was measured using CCK-8 assay. (**K**) Cell proliferation ability was analyzed by EdU assay. (**L**) Flow cytometry for SH-SY5Y cells apoptosis. *n* = 3, *p < 0.05, **p < 0.01, ***p < 0.001

We next explored the protective effect of UBC9 on mitophagy and oxidative stress in SH-SY5Y cells. SH-SY5Y cells were transfected with oe-UBC9 and/or treated with the mitophagy inhibitor CsA, followed by MPP^+^ treatment. As showed in Fig. 2A, treatment with MPP^+^ or MPP^+^ + oe-NC significantly up-regulated the ratio of LC3II/I and p62 expression in the mitochondria and cytosol, and down-regulated PINK1 and Parkin expression in the cytosol of SH-SY5Y cells compared with cells without MPP^+^ stimulation. However, actin in mitochondria and COX IV in cytosol were undetectable. In contrast, overexpression of UBC9 led to the up-regulation of the expression of PINK1, Parkin and the ratio of LC3II/I, and down-regulation of p62 expression, which was reversed by the addition of CsA. The co-localization of mitochondria and autophagosomes in SH-SY5Y cells was also detected by staining with MitoTracker Red and LC3. Our data revealed that the fluorescence intensity of LC3-positive cells in both the MPP^+^ group and the MPP^+^ + oe-NC group was significantly higher than that in the control group (Fig. 2B). Conversely, compared to the MPP^+^ group, overexpression of UBC9 significantly up-regulated LC3 expression in mitochondria in MPP^+^-induced SH-SY5Y cells, and LC3 expression was down-regulated by addition of CsA. TEM revealed that mitochondria in the control group exhibited normal morphology, distinct cristae, and occasional autophagic vesicles. In contrast, MPP⁺-induced mitochondria displayed marked swelling, widened cristae, and an increased number of autophagic vesicles. Overexpression of UBC9 alleviated the MPP⁺-induced mitochondrial dysfunction, an effect that was partially reversed by CsA (Fig. 2C). Furthermore, the results in Fig. 2D suggested that MPP^+^ induction markedly enhanced the JC-1 green fluorescence of SH-SY5Y cells. However, the mitochondrial membrane potential was suppressed by up-regulation of UBC9 and further increased after exposure with CsA (Fig. 2D). The ROS level as detected using DCFH-DA staining in the MPP^+^ and MPP^+^ + oe-NC groups was stronger than that in the control group, while weaker in the MPP^+^ + oe-UBC9 group, as opposed to that in the MPP^+^ group. However, treatment with CsA reversed the effect of UBC9 overexpression (Fig. 2E). Besides, the MPP^+^ group presented increased SOD, CAT, GPX levels and reduced MDA level, whereas up-regulation of UBC9 prevented the effects of MPP^+^ on antioxidant enzyme activities, which was partially reversed by CsA treatment (Fig. 2F-I). Moreover, SH-SY5Y cells proliferation and apoptosis were determined. As presented in Fig. 2J-2K, compared with the control group, treatment with MPP^+^ led to a significant reduction in the proliferation ability of SH-SY5Y cells, as demonstrated by decreased cell viability and reduced EdU-positive cell numbers. Meanwhile, flow cytometry analysis revealed that MPP^+^ treatment notably increased cell apoptosis, in contrast to control group (Fig. 2L). Moreover, SH-SY5Y cells proliferation was notably elevated, while cells apoptosis were inhibited after overexpression of UBC9. CsA partially reversed the effects of UBC9 overexpression on SH-SY5Y cells proliferation and apoptosis. These results collectively suggested that UBC9 regulated MPP^+^-induced proliferation and apoptosis of SH-SY5Y cells by modulating mitophagy to alleviate oxidative stress.

### UBC9 enhanced PINK1 stability by promoting SUMOylation of PINK1

**Fig. 3:**
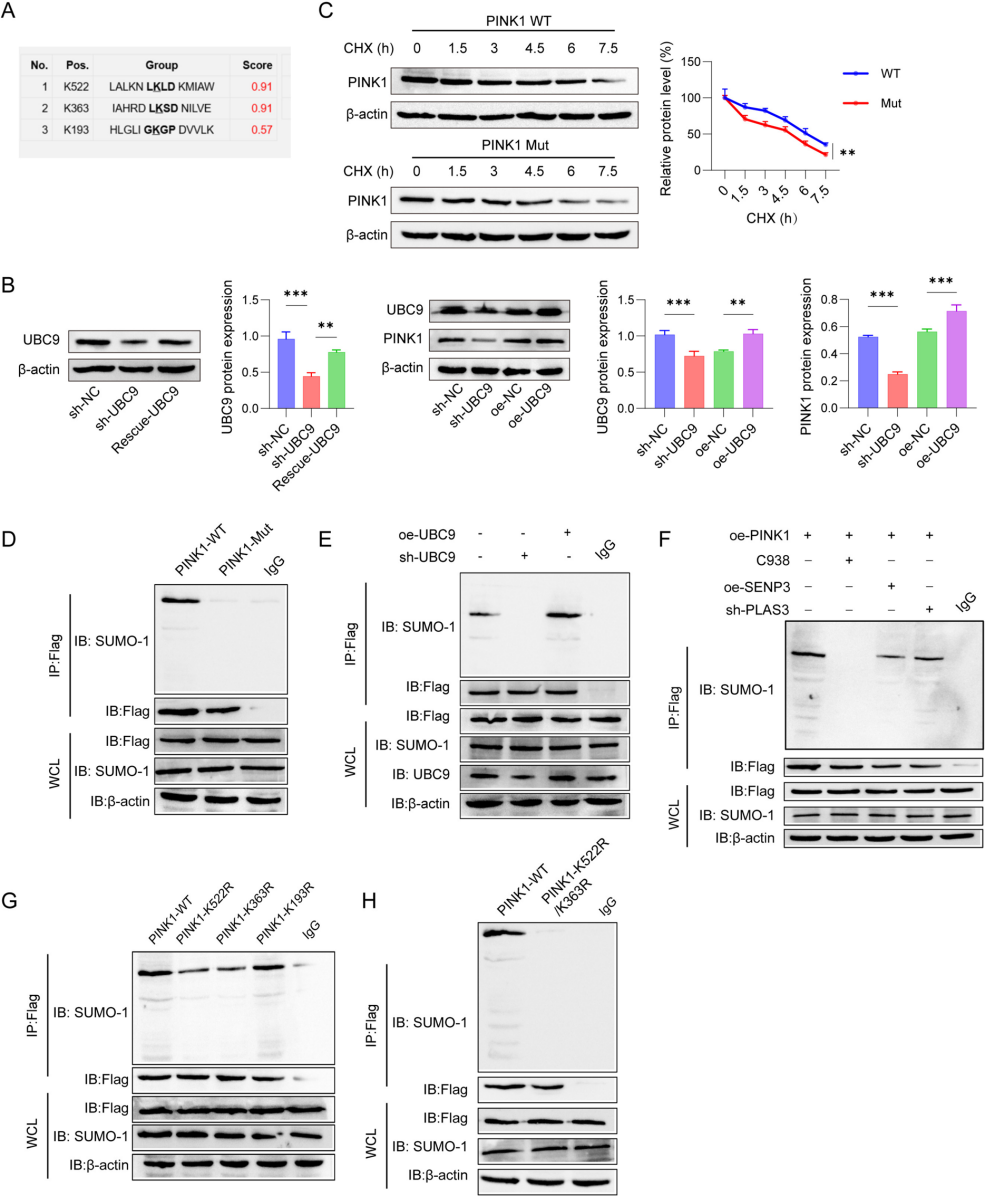
Effects of UBC9 on the SUMOylation of PINK1. (**A**) Potential SUMOylation modification sites in PINK1 were identified by SUMOplot™ Analysis Program. (**B**) The expression of UBC9 and PINK1 were determined using Western blot. (**C**) Degradation rate of the PINK1 protein after CHX chase. (**D**) Western blot for the SUMOylation of PINK1 in SH-SY5Y cells transfected with plasmids encoding Flag-tagged WT PINK1 or PINK1 mutant, together with plasmid encoding SUMO1. (**E**) Western blot analysis was applied to detect the expression of UBC9 and PINK1. (**F**) SUMOylation levels of PINK1 in HEK293T cells transfected with oe-UBC9 or sh-UBC9 were measured using CO-IP assays. (**G**) PINK1 SUMOylation assay using HEK293T cells co-transfected with WT PINK1 or its mutant variants and SUMO1. (**H**) WT PINK1 or K522R/K363R mutant PINK1 was individually transfected with SUMO1 into HEK293T cells for PINK1 SUMOylation assay. *n* = 3, *p < 0.05, **p < 0.01, ***p < 0.001

We next verified whether PINK1 could be modified by UBC9-mediated SUMOylation, lysine (K) residues at positions 522, 363, and 193 as the possible SUMOylation sites in PINK1 by the SUMOplot™ Analysis Program (https://www.abcepta.com/sumoplot) (Fig. 3A). To confirm the transfection efficacy, we assessed UBC9 expression and observed a significant decrease after knockdown and a marked increase after overexpression (Fig. 3B). Subsequently, we assessed the protein half-life in HEK293T cells expressing either wild-type (WT) or a SUMOylation-deficient mutant (Mut) of PINK1. As shown in Fig. 3C, the half-life of Mut PINK1 was significantly shorter than that of WT PINK1, suggesting that SUMOylation may stabilize the PINK1 protein. Western blot further confirmed that PINK1 expression was decreased after knockdown of UBC9 and increased after overexpression of UBC9, and PINK1 stability was increased after overexpression of UBC9 (Fig. 3D). Next, to explore whether UBC9 was responsible for mediating the SUMOylation of PINK1, we knocked down UBC9 by shRNA or overexpressed it in HEK293T cells, followed by SUMOylation analysis. SUMOylation of PINK1 was decreased after knockdown of UBC9 and increased after overexpression of UBC9, indicating that UBC9 mediated SUMOylation of PINK1 (Fig. 3E). Besides, we sought to verify the SUMO modification site(s) in PINK1. Three lysine residues, K522, K363, and K193, were mutated to arginine (R) for SUMOylation identification were constructed. The specificity of PINK1 SUMOylation by targeting distinct nodes of the SUMO pathway was also detected. The SUMOylation of PINK1 was significantly attenuated by the UBC9 mutant (C93S), overexpression of SENP3, and knockdown of PIAS3, indicating that PINK1 SUMOylation is modulated by multiple regulators but depends predominantly on UBC9 (Fig. 3F). As presented in Fig. 3G, the SUMOylation levels of cells transfected with K522R or K363R PINK1 plasmid were notably inhibited in contrast to that of WT PINK1 transfected cells, while K193R showed little impact on PINK1 SUMOylation, revealing that K522 and K363 are likely the SUMOylation sites. Importantly, SUMOylated PINK1 could not be measured in the cells transfected K522R/K363R mutant (Fig. 3H). Our findings suggested that UBC9 promoted PINK1 stability by enhancing SUMOylation of PINK1.

### SUMO1 modification at the PINK1 K522R/K363R position regulates SH-SY5Y cell proliferation and apoptosis

**Fig. 4:**
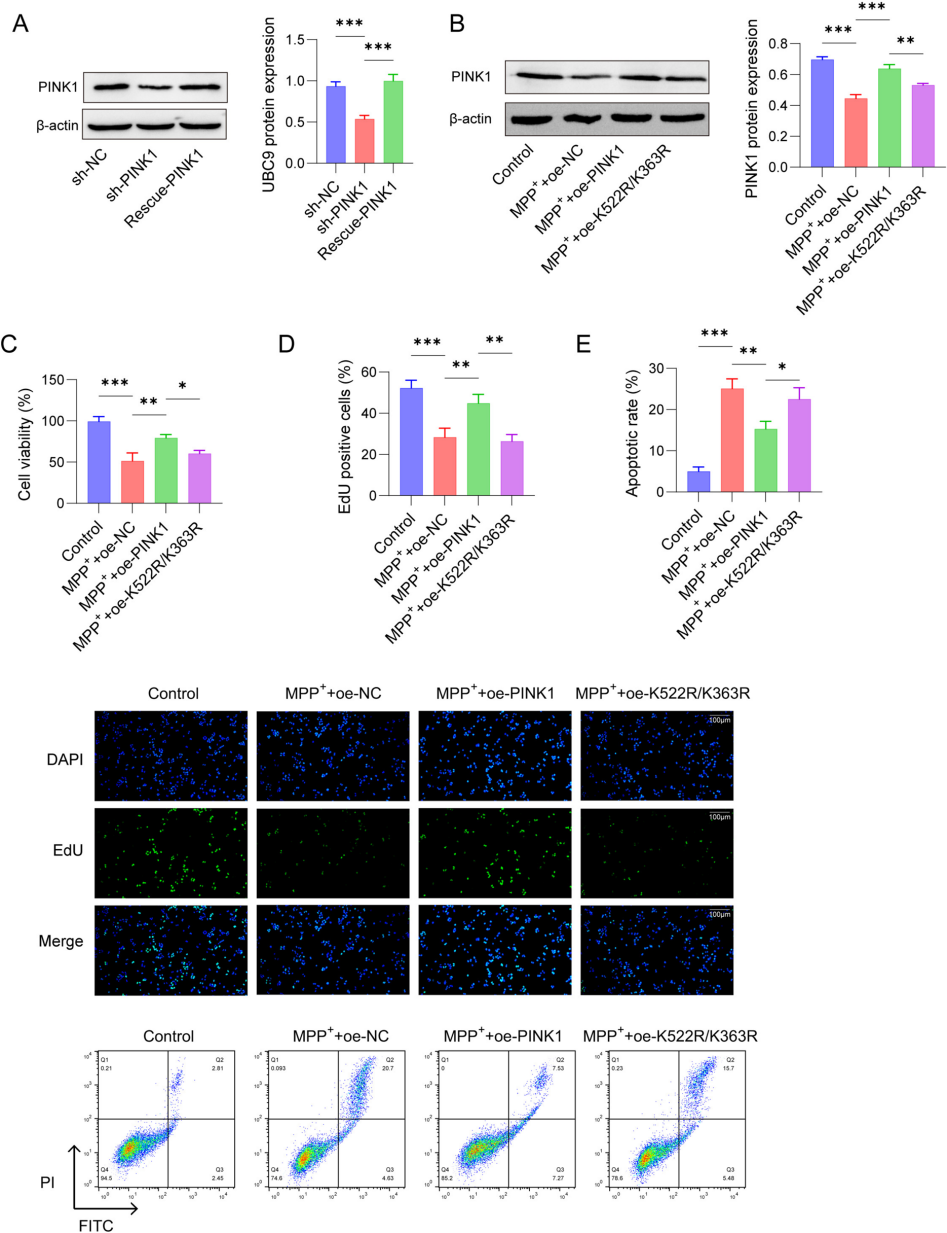
Effects of PINK1 K522R/K363R SUMOylation on MPP^+^-treated SH-SY5Y cells viability and apoptosis. SH-SY5Y cells transfected with oe-PINK1 or oe-PINK1 K522R/K363R were treated with 2 mM MPP.^+^ for 24 h. (**A**) Western blot for protein expression of PINK1. (**B**) CCK-8 for cell viability. (**C**) Cells proliferation was determined by EdU assay. (**D**) Flow cytometry for cell apoptosis. *n* = 3, *p < 0.05, **p < 0.01, ***p < 0.001

To further verify whether SUMO1 modification at the PINK1 K522R/K363R position could regulate SH-SY5Y cell proliferation and apoptosis, SH-SY5Y cells were treated with 1 mM MPP^+^ for 24 h and transfected with oe-PINK1 or oe-PINK1 K522R/K363R plasmid. Western blot demonstrated that PINK1 was significantly down-regulated in MPP^+^-induced SH-SY5Y cells, while up-regulated after overexpression of PINK1. Moreover, PINK1 expression elevated by oe-PINK1 was partly reversed after mutation of PINK1 K522R/K363R (Fig. 4A). In addition, results in Fig. 4B-4D revealed that MPP^+^ treatment led to decreased cell viability and proliferation, and promoted cells apoptosis in SH-SY5Y cells compared with control group. However, in contrast to MPP^+^ + oe-NC group, PINK1 overexpression increased cell viability and proliferation, and reduced apoptotic cells, which was partly abolished in response to mutation PINK1 K522R/K363R. The above results indicated that SUMO1 modification at PINK1 K522R/K363R position could regulate SH-SY5Y cell proliferation and apoptosis.

### UBC9 regulated MPP^+^-stimulated SH-SY5Y cells proliferation and apoptosis via mediating PINK1 SUMOylation

**Fig. 5:**
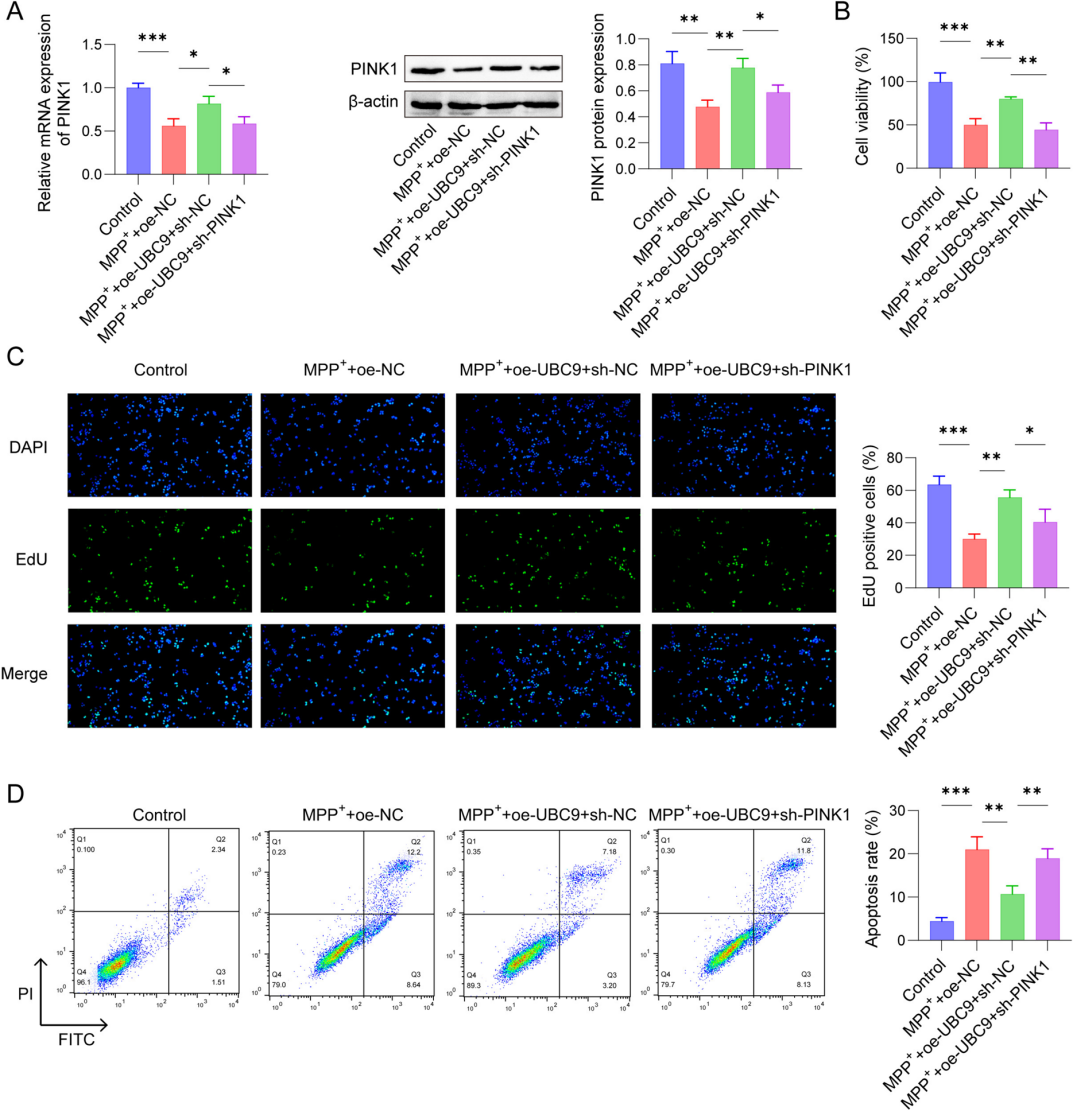
Effects of UBC9 and PINK1 on MPP^+^-treated SH-SY5Y cells viability and apoptosis. SH-SY5Y cells were stimulated by MPP.^+^ with/without transfected with oe-NC or oe-UBC9 or oe-UBC9 + sh PINK1. (**A**) UBC9 expression was measured using Western blot. (**B**) PINK1 expression and mRNA levels were measured using Western blot or qRT-PCR. (**C**) Cell viability was measured using CCK-8 assay. (**D**) EdU assay was applied to detect cell proliferation ability. (**E**) Flow cytometry for SH-SY5Y cells apoptosis. *n* = 3, *p < 0.05, **p < 0.01, ***p < 0.001

To evaluate if UBC9 was involved in SH-SY5Y cells proliferation and apoptosis by regulating PINK1, we overexpressed UBC9 or/and silenced PINK1 in MPP^+^-stimulated cells. We confirmed the efficacy of transfection, as expected, knockdown of PINK1 decreased PINK1 protein level, whereas PINK1 overexpression increased it (Fig. 5A). We also found that UBC9 overexpression increased PINK1 expression in the MPP^+^-induced cells compared to the MPP^+^ + oe-NC group, while obviously inhibition was seen after silencing PINK1 (Fig. 5B). CCK-8 assay further suggested that MPP^+^ induction remarkably inhibited cell survival of SH-SY5Y cells, which was counteracted by up-regulation of UBC9. However, the enhanced SH-SY5Y cells viability induced by UBC9 overexpression was suppressed after PINK1 silence (Fig. 5C). Besides, compared with the control group, MPP^+^ + oe-NC treatment decreased cells proliferation and increased the apoptotic cells. Similarly, under MPP^+^ + oe-NC conditions, overexpression of UBC9 enhanced the proliferation ability of SH-SY5Y and suppressed the apoptosis ratio, which was notably rescued by PINK1 knockdown (Fig. 5D-E). Our findings demonstrated that UBC9 promoted MPP^+^-induced cells proliferation and reduced cells apoptosis via regulating PINK1 SUMOylation.

### UBC9 attenuated oxidative stress of MPP^+^-stimulated SH-SY5Y cells via PINK1-mediated mitophagy

**Fig. 6:**
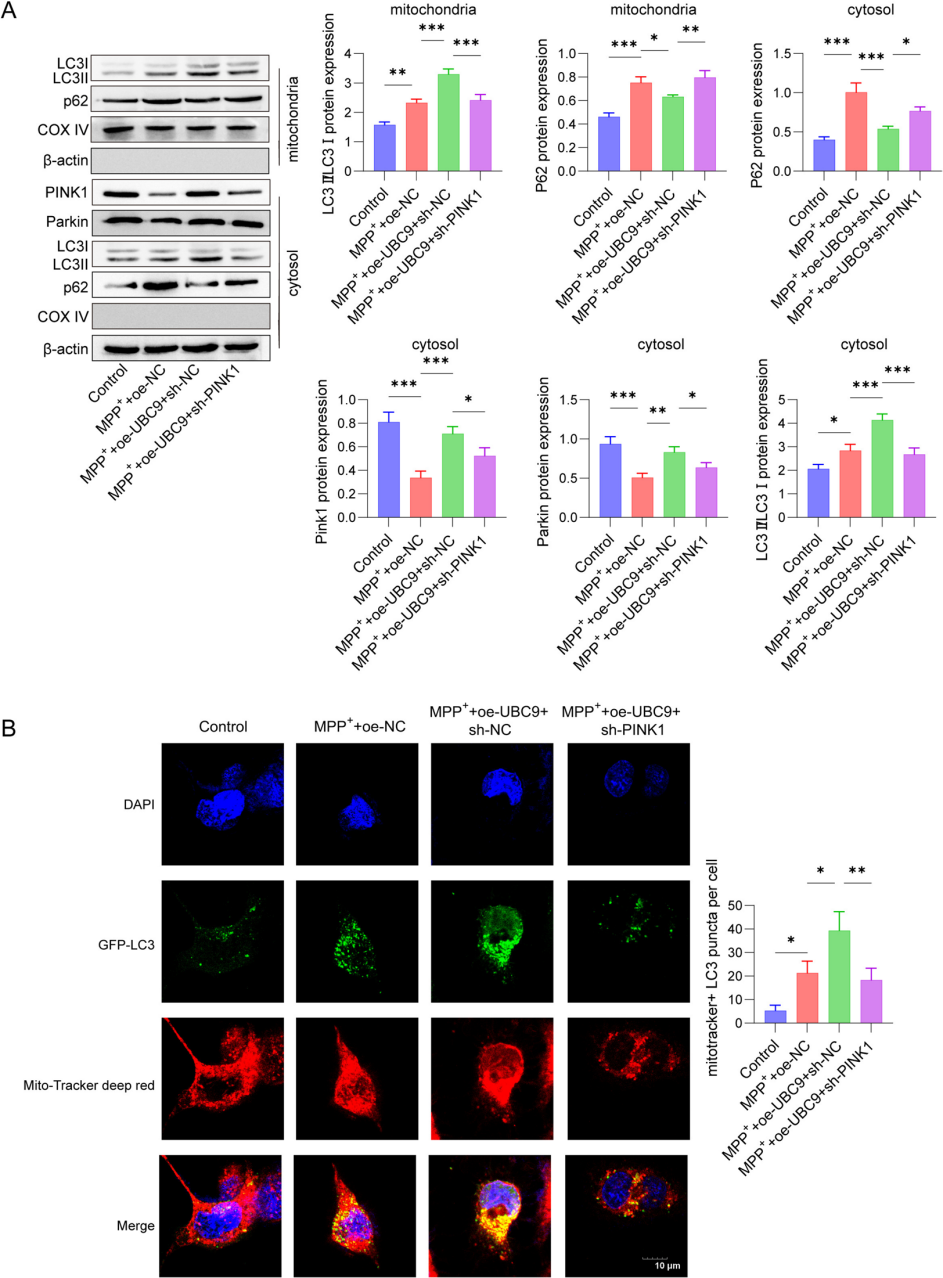

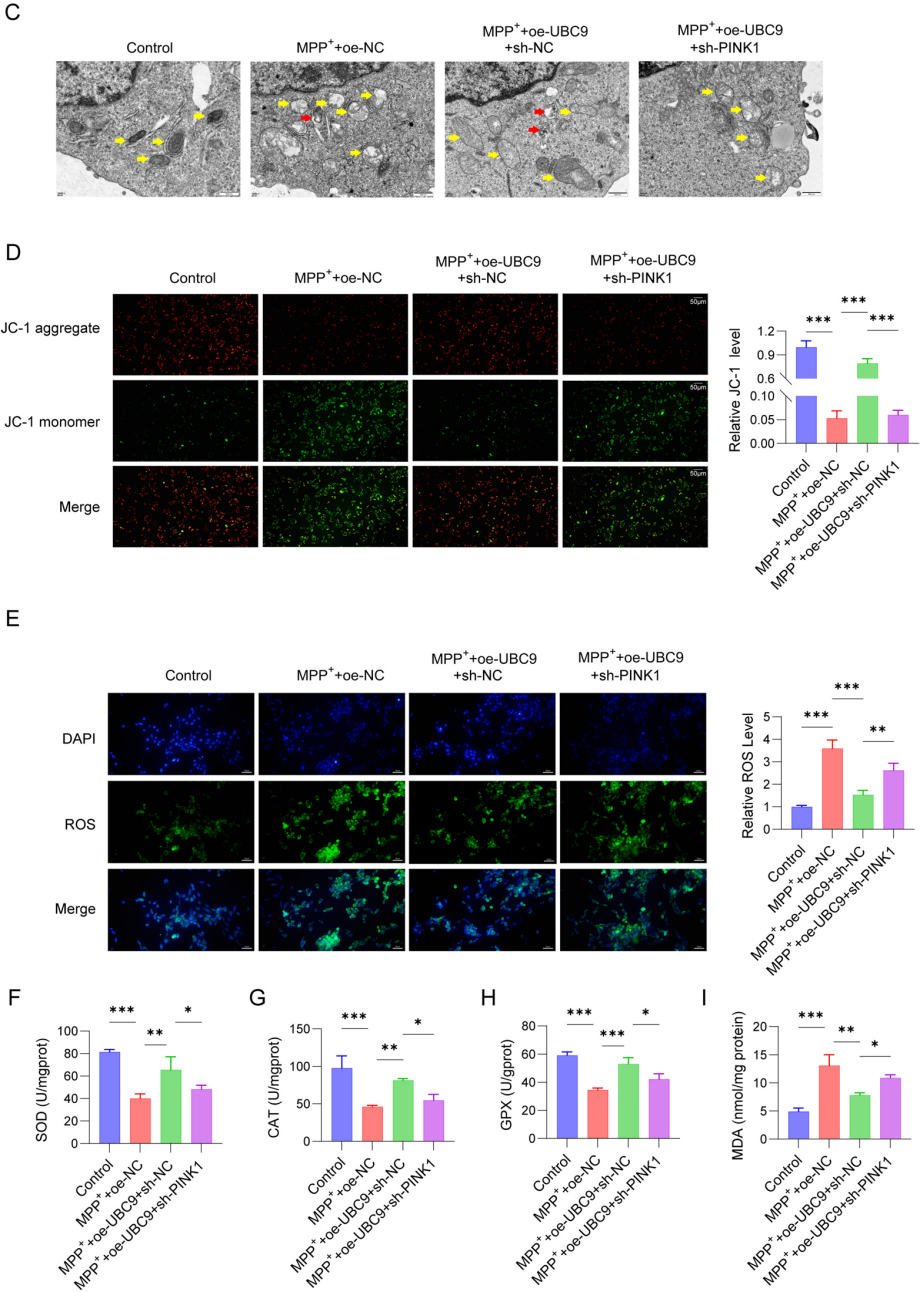
Effects of UBC9 and PINK1 on oxidative stress and mitophagy in MPP^+^-induced SH-SY5Y cells. SH-SY5Y cells transfected with oe-NC, oe-UBC9 + sh NC or oe-UBC9 + sh PINK1 were treated with 2 mM MPP.^+^ for 24 h. (**A**) Western blot for autophagy-related proteins. (**B**) Immunofluorescence detection of LC3 content in mitochondria. (**C**) Representative TEM images of mitochondria. (**D**) The mitochondrial membrane potential changes were assessed using JC-1 staining. (**E**) ROS generation was evaluated by DCFH-DA staining. (**F**-**I**) Enzyme activities of SOD, CAT, Gpx and MDA were determined. *n* = 3, *p < 0.05, **p < 0.01, ***p < 0.001

We further illustrated whether UBC9/PINK1 contributed to the oxidative stress of MPP^+^-induced cells. Data from western blot reveal a higher p62 and LC3II/I expression in the mitochondria of SH-SY5Y cells in MPP^+^ + oe-NC group than in the control group. Meanwhile, the p62 and LC3II/I expression was enhanced, while PINK1 and Parkin expression was reduced in the cytosol of SH-SY5Y cells in the MPP^+^ + oe-NC group. Moreover, actin in mitochondria and COX IV in cytosol were undetectable. Overexpression of UBC9 further up-regulated the expression of LC3II/I, PINK1, Parkin and suppressed the p62 expression, which was reversed after PINK1 silence (Fig. 6A). Results of immunofluorescence showed that the fluorescence intensity of LC3-positive cells in both the MPP^+^ group and the MPP^+^ + oe-NC group was significantly higher than that in the control group. Overexpression of UBC9 significantly up-regulated mitochondrial LC3 expression in MPP^+^-induced SH-SY5Y cells, whereas the LC3 expression was down-regulated after knocking down PINK1 (Fig. 6B). TEM revealed that control cells exhibited normal mitochondrial morphology, characterized by intact cristae and the occasional presence of autophagic vesicles. In contrast, MPP⁺ treatment induced severe mitochondrial swelling, cristae widening, and a marked increase in autophagic vesicles. Overexpression of UBC9 notably attenuated these MPP⁺-induced ultrastructural abnormalities. However, this protective effect was partially reversed upon PINK1 knockdown (Fig. 6C). Moreover, JC-1 staining demonstrated that the JC-1 green fluorescence of SH-SY5Y cells were markedly inhibited in MPP^+^ + oe-NC group. However, after co-cultured with MPP^+^, UBC9 overexpression increased the JC-1 green fluorescence, which was reversed after PINK1 silence (Fig. 6D). DCFH-DA staining revealed that MPP^+^ + oe-NC had the stronger ROS level than that in the control group, while UBC9 overexpression inhibited ROS level, which was abrogated by PINK1 silence (Fig. 6E). In addition, MPP^+^ + oe-NC treatment obviously suppressed SOD, CAT, GPX levels and enhanced MDA level. We observed the opposite results in UBC9 overexpression group, and the antioxidant enzyme activities were partially reversed by PINK1 silence (Fig. 6F-I). These findings indicated that UBC9 alleviated MPP^+^-induced oxidative stress via regulating PINK1-mediated mitophagy.

### UBC9 modulated mitophagy in PD mice to alleviate oxidative stress by mediating SUMOylation of PINK1

**Fig. 7:**
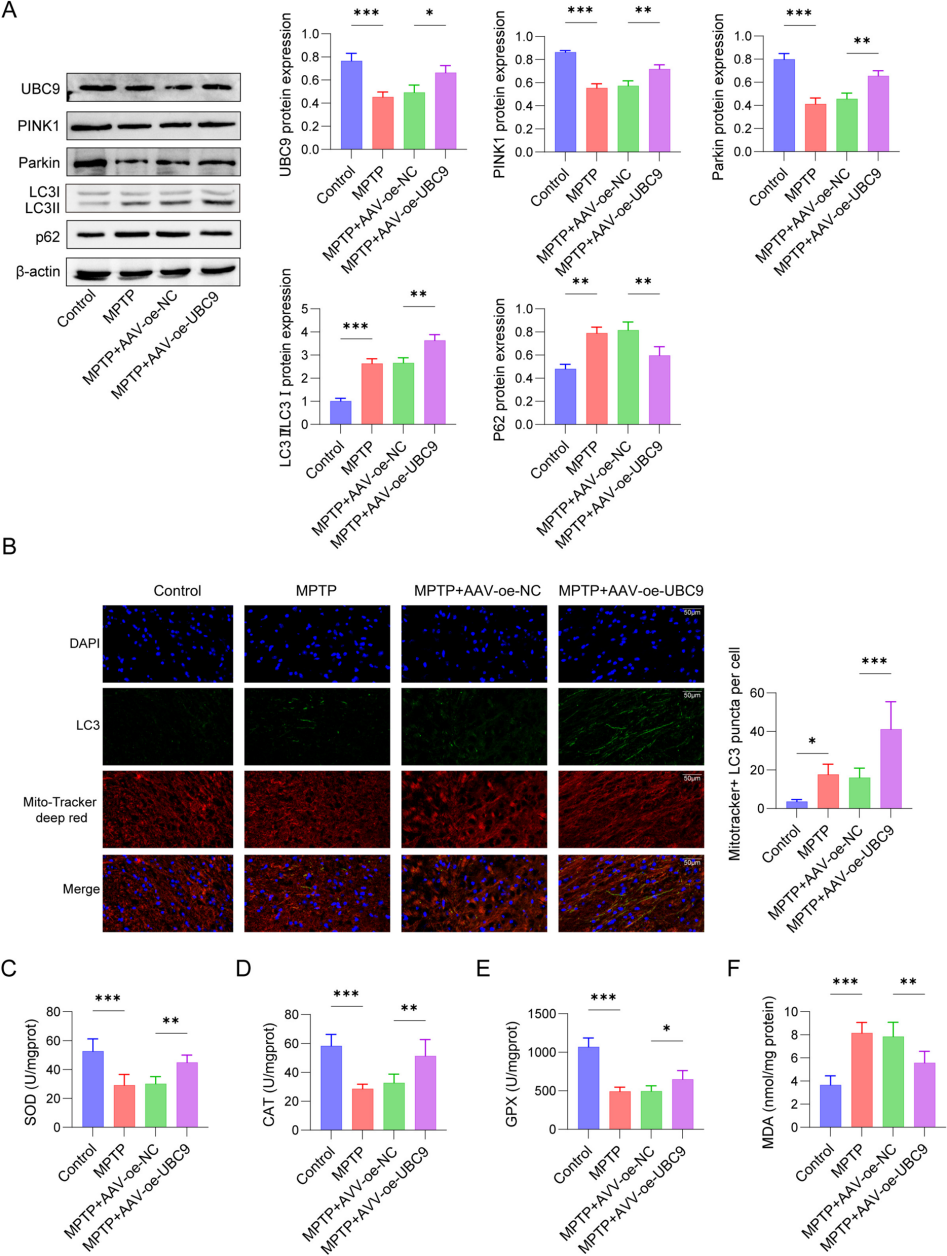
Effect of UBC9 on oxidative stress and mitophagy in MPTP-induced mice. PD mice were given AVV-oe NC or AVV-oe UBC9 and injected intraperitoneally (i.p.) with MPTP (25 mg/kg/day). (**A**) Western blot for autophagy-related proteins. (**B**) Representative immunofluorescence images of LC3. (C-F) Enzyme activities of SOD, CAT, Gpxand MDA were determined. *n* = 3, *p < 0.05, **p < 0.01, ***p < 0.001

Although UBC9 regulates mitophagy to attenuate oxidative stress by mediating SUMOylation of PINK1 in *vitro*, further study is required to explore the function of UBC9 in *vivo*. UBC9 overexpression was conducted by AAV vectors (AAV -oe-UBC9) in a MPTP -induced mouse model. As presented in Fig. 7A, MPTP treatment led to a decreased in UBC9, PINK1 and Parkin expression, and an increased in LC3 and p62 expression in PD mice. However, MPTP + AAV -oe-UBC9 up-regulated UBC9, PINK1, Parkin, and LC3 expression, and down-regulated p62 expression in mice, compared to MPTP + AAV -oe-NC group. Moreover, the fluorescence intensity of LC3-positive cells in both the MPTP group and MPTP + AAV -oe-NC group was remarkably higher than that in the control group. Overexpression of UBC9 further enhanced mitochondrial LC3 expression in PD mice (Fig. 7B). To further validate the modulation of oxidative stress in PD, we detected several oxidative stress-associated indicators. MPTP induction significantly reduced SOD, CAT and GPX levels, but markedly increased MDA levels in MPTP mice, whereas overexpression of UBC9 partially reversed the effects of MPTP on oxidative stress (Fig. 7C-7F). These observations demonstrated that UBC9 modulated mitophagy in PD mice to alleviate oxidative stress by mediating SUMOylation of PINK1.

### UBC9 alleviated MPTP-induced motor dysfunction in PD

**Fig. 8:**
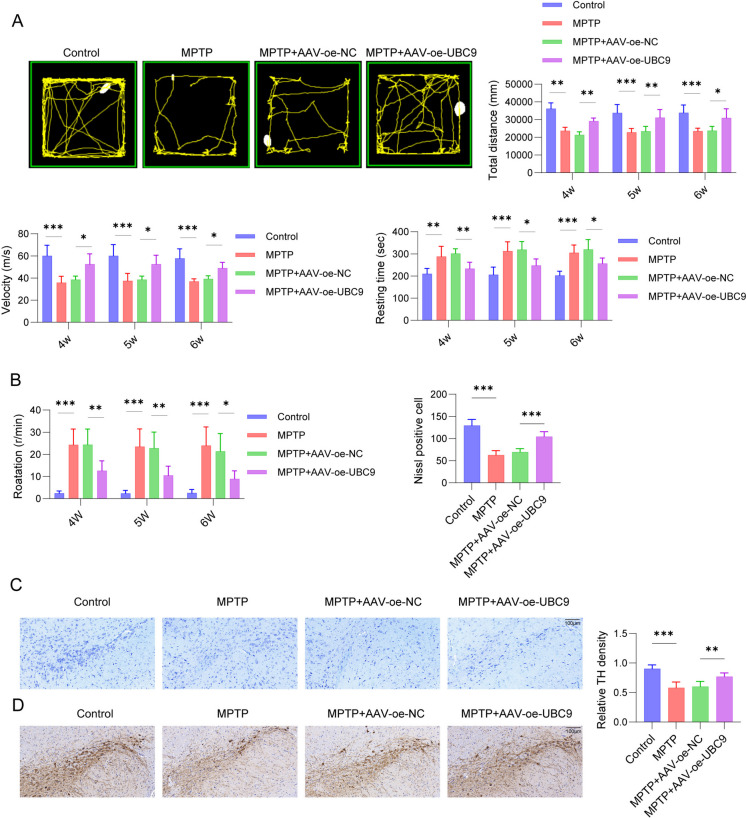
Effect of UBC9 on motor dysfunction of MPTP-induced PD mice. PD mice were given AVV-oe NC or AVV-oe UBC9 and injected intraperitoneally (i.p.) with MPTP (25 mg/kg/day). (**A**) Mice motor dysfunction in the total distance moved test, velocity test and resting time test. (**B**) Latency to fall and pole-climbing time were calculated. (**C**) Nissl staining was applied to assess the brain substantia nigra region of the mice. (*D*) TH expression was evaluated using IHC staining. *n* = 3, *p < 0.05, **p < 0.01, ***p < 0.001

To further assess the influence of UBC9 on MPTP-treated motor dysfunction, the behavioral function of PD mice was assessed. Figure 8A displayed that MPTP-induced mice displayed an obvious decrease in open field activity, in construct to control group, while UBC9 overexpression remarkably enhanced locomotor activity. The statistical analysis indicated that MPTP resulted in a reduced in the total distance moved, velocity, a prolongation of resting time and lower APO rotation scores,whereas UBC9 overexpression partially reverses the effects of MPTP induction on the above behaviors. Results in Fig. 8B revealed that MPTP-induced mice had significantly reduced latency to fall and increased pole-climbing time, whereas overexpressing UBC9 reversed these results. Nissl staining results demonstrated that in the normal group, the neurons of mice exhibited an intact structure, with normal cell morphology, clear boundaries of the cell nucleus, and visible blue-colored Nissl bodies. In contrast, in the MPTP group, there was a reduction in the number of neurons, alterations in cell morphology and structure, and a decrease in the number of Nissl-positive cells. When compared with the MPTP + AAV -oe-NC group, the MPTP + AAV -oe-UBC9 group showed a significant improvement in neuron morphology and structure, increased neurons and Nissl-body-positive cells number (Fig. 8C). Furthermore, the number of substantia nigra TH-positive neurons was notably decreased in the MPTP-treated mice, which was enhanced by UBC9 overexpression (Fig. 8D). These findings above indicated that UBC9 ameliorated motor dysfunction of MPTP-treated mice.

### UBC9 attenuates PD neurotoxicity by promoting PINK1 SUMOylation to enhance mitophagy and reduce oxidative stress

**Fig. 9:**
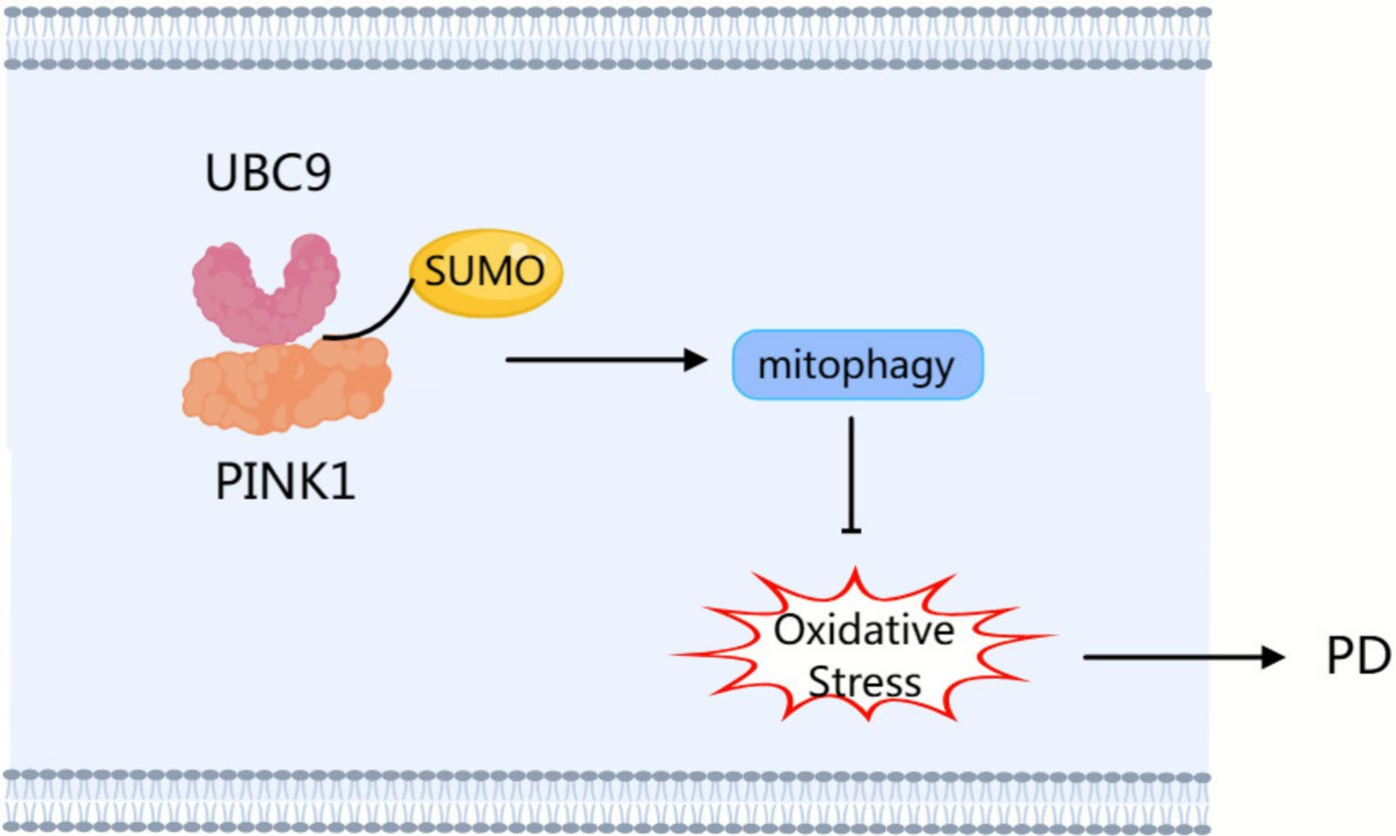
The schematic model illustrates the proposed neuroprotective mechanism of UBC9 in cellular and mouse models of PD

This study elucidates a novel neuroprotective pathway in which UBC9 plays a central role in counteracting PD-related neurotoxicity. We demonstrate that (1) UBC9 catalyzes the SUMOylation of PINK1 at key lysine residues (K363 and K522), which enhances PINK1 protein stability. (2) UBC9-mediated PINK1 SUMOylation activates the PINK1/Parkin pathway, thereby promoting mitophagy and alleviating mitochondrial dysfunction. (3) The UBC9/PINK1 axis mitigates oxidative stress. (4) Overexpression of UBC9 confers neuroprotection, rescues dopaminergic neurons, and ameliorates motor deficits in PD models. In summary, our findings demonstrate that UBC9-mediated SUMOylation of PINK1 stabilizes the PINK1 protein, thereby augmenting mitophagy and alleviating oxidative stress, which ultimately confers a protective effect against the pathogenesis of PD (Fig. 9).

## Discussion

Parkinson’s disease is a common neurodegenerative disease that occurs in the elderly. The pathology of PD is featured with the progressive loss of nigrostriatal dopaminergic neurons in the brain and the accumulation of toxic proteins in Lewy vesicles (Morris et al. 2024). A growing number of researches indicated the pathogenesis of PD is related to apoptosis, autophagy and mitochondrial dysfunction (Liang et al. 2024). UBC9, a kind of SUMO E2-binding enzyme, was reported to regulate multiple diseases (Xiao et al. 2023). Our study reported for the first time that UBC9 regulates SUMOylation of PINK1 to relieve oxidative stress and block the progression of PD. Importantly, the potential mechanism of oxidative stress inhibition may be derived from the SUMO-mediated mitophagy induced by MPTP or MPP^+^. These findings position UBC9 as a critical regulator within the PD pathophysiological landscape.MPP^+^ was the neurotoxic form of methyl 4 phenyl 1,2,3,6 tetrahydropyridine (MPTP), which causes mitochondrial dysfunction, oxidative stress and programmed cell death after uptake by dopaminergic neurons (Vaidya et al. 2023). Song et al. reported that baicalein protects against MPP^+^/MPTP-induced neurotoxicity via relieving oxidative stress in PD (Song et al. 2021). In this research, We observed that MPP⁺/MPTP exposure significantly downregulated UBC9 expression, consistent with previous findings that UBC9 protects dopaminergic cells from cytotoxicity (Verma et al. 2020). Importantly, overexpression of UBC9 reversed the inhibitory effect of MPP^+^/MPTP on UBC9 expression, cell viability and apoptosis, and ameliorated motor deficits in mice. Concurrently, the overexpression of UBC9 elevated tyrosine hydroxylase (TH) levels in the substantia nigra, further supporting its role in preserving dopaminergic neuron function. These consistent findings across *in vitro* and *in vivo* systems strongly indicate that UBC9 acts as a critical regulatory factor against parkinsonian neurotoxicity. However, the central role of UBC9 as the sole SUMO E2 conjugating enzyme necessitates a cautious interpretation of its therapeutic potential. Wang et al. suggested that UBC9 overexpression promotes proliferation and metastasis of gastric cancer by ATF2 (Wang et al. 2025). Besides, UBC9 upregulation by RREB1 underlies enhanced SUMOylation and chemoresistance in colorectal cancer (Deng et al. 2024). Therefore, systemic or long-term upregulation of UBC9 carries an inherent risk of off-target effects, such as carcinogenesis. Our data, while promising, highlight the necessity for future research to develop strategies for targeted UBC9 activation. Such strategies could include the identification of small-molecule agonists that enhance the specific UBC9-PINK1 interaction or the development of neuron-specific delivery systems to spatially constrain UBC9 activity, thereby mitigating risks associated with its broad functions.

Increasing researches suggested that autophagy dysfunction is linked with the pathogenesis of several neurodegenerative diseases, including PD. For example, Wang et al. found that lidocaine promoted SH-SY5Y cells autophagy by inactivation of PI3K/AKT/mTOR pathway and regulating miR-145 (Wang et al. 2020). In addition, exosomes derived from mesenchymal stem cells blocked the development of PD by inducing autophagy (Chen et al. 2020). Prior researches have also reported the influence of UBC9 on autophagy (Fan et al. 2024). In this investigation, overexpression of UBC9 reversed the MPP^+^-induced elevation of p62 expression, the reduction of PINK1 and Parkin expression and further promoted the LC3 expressionin SH-SY5Y cells, which was reversed by addition of CsA. These data are similar to those previously reported by Chen et al., suggesting that UBC9 promoted UBC9 could prevent PD progression through promoting cell autophagy (Chen et al. 2022). Consistent with the cellular model, UBC9 overexpression in MPTP-induced mice also restored the levels of autophagy-related protein and increased TH-positive neurons. Oxidative stress plays an extremely important part in the development of PD. Oxidative stress-induced apoptosis is involved in the progression of neurological diseases, and over production of ROS induces oxidative stress has been implicated in neuronal death (Zhu et al. 2024). Jiang et al. suggested that Apolipoprotein A-I mimetic peptides enhanced antioxidant properties via promoting the activities of SOD, CAT and GSH-Px, and suppressing MDA concentration (Jiang and Bai 2022). Similar to this report, our data suggested that overexpression of UBC9 reversed the features of MPP^+^/MPTP-induced oxidative stress, including elevated mitochondrial membrane potential, SOD and GSH levels, and reduced ROS and MDA levels, which was reversed by CsA. Collectively, our findings position UBC9 as a potential therapeutic target for PD. In neurodegenerative diseases, SUMOylation has emerged as a key player in regulating relevant proteins expression, including tau proteins and α-synuclein (Savyon and Engelender 2020). Previous researches have showed that SUMOylation plays an important role in regulating mitophagy activation and protects cells from oxidative stress by reducing ROS production (Soares et al. 2024). Here, we identified PINK1 as a substrate for UBC9-regulated SUMOylation. PINK1, a protein associated with PD, regulates mitophagy by recruiting the E3 ubiquitin ligase Parkin (Malpartida et al. 2021; Jiang et al. 2024). Our study predicted for the first time the SUMOisation site of PINK1 and UBC9 enhanced PINK1 stability by promoting SUMOylation at K522R, K363R and K193R sites. More importantly, PINK1 silence partly abolished the influence of UBC9 overexpression on proliferation, apoptosis, mitophagy and oxidative stress in PD. In summary, our results elucidated that UBC9 attenuated oxidative stress via PINK1-mediated mitophagy in PD, highlighting UBC9 as a potential diagnostic biomarker and therapeutic target.Taken together, our results revealed the protective effect of UBC9 against MPTP-induced neurotoxicity in vitro and in vivo. Besides, the neuroprotective effect of UBC9 may be related to the promotion of SUMOylation of PINK1, the enhance of mitophagy and the reduction of oxidative stress. UBC9 was identified as a promising player in the pathophysiological process of PD. However, our investigation has several limitations. First, K522 and K363 are bona fide SUMOylation sites is primarily supported by predictive software and co-immunoprecipitation assays. Mass spectrometry or site-specific functional assays is required to provide unequivocal evidence and to delineate the functional consequences of these modifications on mitophagy and neuronal survival. Second, whether UBC9 exerts its protective role in our MPTP/MPP⁺-induced models by regulating other critical pathways needs further investigation. Third, the acute neurotoxin models do not fully recapitulate the chronic, progressive proteinopathy characteristic of human PD. Chronic models, such as those utilizing α-synuclein pre-formed fibrils (PFF) or transgenic mice, will be crucial to validate and extend our findings.

## Data Availability

The datasets used or analyzed during the current study are available from the corresponding author on reasonable request.

## Abbreviations

ATCC: American Type Culture Collection
CCK-8: Cell Counting Kit-8
Co-IP: Co-immunoprecipitation
CsA: Cyclosporin A
DAPI: 4’,6-Diamidino-2-phenylindole
EdU: 5-Ethynyl-2’-deoxyuridine
FBS: Fetal bovine serum
FITC: Fluorescein isothiocyanate
GSH: Glutathione
MDA: Malondialdehyde
MPTP: Methyl 4 phenyl 1,2,3,6 tetrahydropyridine
PD: Parkinson’s disease
PI: Propidium iodide
PINK1: PTEN induced putative kinase 1
qRT-PCR: Quantitative real-time PCR
ROS: Reactive oxygen species
SOD: Superoxide Dismutase
SUMO: Small ubiquitin-like modifier
TH +: Tyrosine hydroxylase
UBC9: SUMO-conjugating enzyme UBC9

## References

[CR1] Chen HX, Liang FC, Gu P, Xu BL, Xu HJ, Wang WT, et al. Exosomes derived from mesenchymal stem cells repair a Parkinson’s disease model by inducing autophagy. Cell Death Dis. 2020;11(4):288. 32341347 10.1038/s41419-020-2473-5 PMC7184757

[CR2] Chen C, Chen Y, Liu T, Song D, Ma D, Cheng O. Dexmedetomidine can enhance PINK1/Parkin-mediated mitophagy in MPTP-induced PD mice model by activating AMPK. Oxid Med Cell Longev. 2022;2022:7511393. 35528513 10.1155/2022/7511393 PMC9068320

[CR3] Deng YN, Chen Y, Gao S, Zhang N, Luo Y, Luo S, et al. RREB1-mediated SUMOylation enhancement promotes chemoresistance partially by transcriptionally upregulating UBC9 in colorectal cancer. Front Pharmacol. 2024;15:1381860. 39108750 10.3389/fphar.2024.1381860 PMC11300207

[CR4] Fan YP, Lou JS, Jin MR, Zhou CH, Shen HH, Fu CY, et al. UBC9-mediated SUMOylation of Lamin B1 enhances DNA-damage-induced nuclear DNA leakage and autophagy after spinal cord injury. J Cell Physiol. 2024;239(5):e31213. 38308641 10.1002/jcp.31213

[CR5] Jiang H, Bai X. Apolipoprotein A-I mimetic peptides (ApoAI MP) improve oxidative stress and inflammatory responses in Parkinson’s disease mice. Front Pharmacol. 2022;13:966232. 36059954 10.3389/fphar.2022.966232 PMC9437339

[CR6] Jiang C, Li Y, Duan K, Zhan T, Chen Z, Wang Y, et al. Parkin deletion affects PINK1/Parkin-mediated mitochondrial autophagy to exacerbate neuroinflammation and accelerate progression of Parkinson’s disease in mice. Nan Fang Yi Ke Da Xue Xue Bao. 2024;44(12):2359–66. 39725624 10.12122/j.issn.1673-4254.2024.12.11 PMC11683357

[CR7] Li A, Gao M, Liu B, Qin Y, Chen L, Liu H, et al. Mitochondrial autophagy: molecular mechanisms and implications for cardiovascular disease. Cell Death Dis. 2022;13(5):444. 35534453 10.1038/s41419-022-04906-6 PMC9085840

[CR8] Liang H, Ma Z, Zhong W, Liu J, Sugimoto K, Chen H. Regulation of mitophagy and mitochondrial function: natural compounds as potential therapeutic strategies for Parkinson’s disease. Phytother Res. 2024;38(4):1838–62. 38356178 10.1002/ptr.8156

[CR9] Malpartida AB, Williamson M, Narendra DP, Wade-Martins R, Ryan BJ. Mitochondrial dysfunction and mitophagy in Parkinson’s disease: from mechanism to therapy. Trends Biochem Sci. 2021;46(4):329–43. 33323315 10.1016/j.tibs.2020.11.007

[CR10] Morris HR, Spillantini MG, Sue CM, Williams-Gray CH. The pathogenesis of Parkinson’s disease. Lancet. 2024;403(10423):293–304. 38245249 10.1016/S0140-6736(23)01478-2

[CR11] Okitsu M, Sugaya K, Nakata Y, Kawazoe T, Ikezawa J, Okiyama R, et al. Degeneration of nigrostriatal dopaminergic neurons in the early to intermediate stage of dementia with Lewy bodies and Parkinson’s disease. J Neurol Sci. 2023;449:120660. 37084522 10.1016/j.jns.2023.120660

[CR12] Picca A, Guerra F, Calvani R, Romano R, Coelho-Júnior HJ, Bucci C, et al. Mitochondrial dysfunction, protein misfolding and neuroinflammation in Parkinson’s disease: roads to biomarker discovery. Biomolecules. 2021. 10.3390/biom11101508. 34680141 10.3390/biom11101508 PMC8534011

[CR13] Prasertsuksri P, Kraokaew P, Pranweerapaiboon K, Sobhon P, Chaithirayanon K. Neuroprotection of andrographolide against neurotoxin MPP(+)-induced apoptosis in SH-SY5Y cells via activating mitophagy, autophagy, and antioxidant activities. Int J Mol Sci. 2023. 10.3390/ijms24108528. 37239873 10.3390/ijms24108528 PMC10217882

[CR14] Qin Y, Li Q, Liang W, Yan R, Tong L, Jia M, et al. TRIM28 sumoylates and stabilizes NLRP3 to facilitate inflammasome activation. Nat Commun. 2021;12(1):4794. 34373456 10.1038/s41467-021-25033-4 PMC8352945

[CR15] Quinn PMJ, Moreira PI, Ambrósio AF, Alves CH. PINK1/PARKIN signalling in neurodegeneration and neuroinflammation. Acta Neuropathol Commun. 2020;8(1):189. 33168089 10.1186/s40478-020-01062-w PMC7654589

[CR16] Sahin U, de Thé H, Lallemand-Breitenbach V. Sumoylation in physiology, pathology and therapy. Cells. 2022. 10.3390/cells11050814. 35269436 10.3390/cells11050814 PMC8909597

[CR17] Savyon M, Engelender S. Sumoylation in α-synuclein homeostasis and pathology. Front Aging Neurosci. 2020;12:167. 32670048 10.3389/fnagi.2020.00167 PMC7330056

[CR18] Simon DK, Tanner CM, Brundin P. Parkinson disease epidemiology, pathology, genetics, and pathophysiology. Clin Geriatr Med. 2020;36(1):1–12. 31733690 10.1016/j.cger.2019.08.002 PMC6905381

[CR19] Soares ES, Queiroz LY, Gerhardt E, Prediger RDS, Outeiro TF, Cimarosti HI. SUMOylation modulates mitochondrial dynamics in an in vitro rotenone model of Parkinson’s disease. Mol Cell Neurosci. 2024;131:103969. 39260456 10.1016/j.mcn.2024.103969

[CR20] Song Q, Peng S, Zhu X. Baicalein protects against MPP(+)/MPTP-induced neurotoxicity by ameliorating oxidative stress in SH-SY5Y cells and mouse model of Parkinson’s disease. Neurotoxicology. 2021;87:188–94. 34666128 10.1016/j.neuro.2021.10.003

[CR21] Vaidya B, Polepalli M, Sharma SS, Singh JN. 2-aminoethoxydiphenyl borate ameliorates mitochondrial dysfunctions in MPTP/MPP(+) model of Parkinson’s disease. Mitochondrion. 2023;69:95–103. 36758857 10.1016/j.mito.2023.02.003

[CR22] Verma DK, Ghosh A, Ruggiero L, Cartier E, Janezic E, Williams D, et al. The SUMO conjugase Ubc9 protects dopaminergic cells from cytotoxicity and enhances the stability of α-synuclein in Parkinson’s disease models. eNeuro. 2020. 10.1523/ENEURO.0134-20.2020. 32887693 10.1523/ENEURO.0134-20.2020 PMC7519168

[CR23] Wang R, Shih LC. Parkinson’s disease - current treatment. Curr Opin Neurol. 2023;36(4):302–8. 37366218 10.1097/WCO.0000000000001166

[CR24] Wang Z, Liu Q, Lu J, Cao J, YWang X, Chen Y. Lidocaine promotes autophagy of SH-SY5Y cells through inhibiting PI3K/AKT/mTOR pathway by upregulating miR-145. Toxicol Res. 2020;9(4):467–73. 10.1093/toxres/tfaa049 PMC7467247 32905277

[CR25] Wang Q, Li S, Xu Y, Chen Y, Xu C, He Q, et al. UBC9 overexpression promotes proliferation and metastasis in gastric cancer via ATF2. World J Surg Oncol. 2025;23(1):270. 40635031 10.1186/s12957-025-03922-y PMC12243259

[CR26] Xiao J, Sun F, Wang YN, Liu B, Zhou P, Wang FX, et al. UBC9 deficiency enhances immunostimulatory macrophage activation and subsequent antitumor T cell response in prostate cancer. J Clin Invest. 2023. 10.1172/JCI158352. 36626227 10.1172/JCI158352 PMC9927932

[CR27] Xu T, Dong W, Liu J, Yin P, Wang Z, Zhang L, et al. Disease burden of Parkinson’s disease in China and its provinces from 1990 to 2021: findings from the global burden of disease study 2021. The Lancet Regional Health. 2024;46:101078. 38745974 10.1016/j.lanwpc.2024.101078 PMC11091691

[CR28] Zhu L, Li Z, Sheng L, Zhang F, Ji W. Ginkgolide A attenuated apoptosis via inhibition of oxidative stress in mice with traumatic brain injury. Heliyon. 2024;10(2):e24759. 38304806 10.1016/j.heliyon.2024.e24759 PMC10830544

